# The relationship between cell density and cell count differs among *Saccharomyces* yeast species

**DOI:** 10.17912/micropub.biology.001215

**Published:** 2024-05-23

**Authors:** Javier Pinto, Nik Tavakolian, Chun-Biu Li, Rike Stelkens

**Affiliations:** 1 Zoology Department, Stockholm University, Stockholm, Sweden; 2 Department of Mathematics, Stockholm University, Stockholm, Sweden

## Abstract

There is a recent push to develop wild and non-domesticated *Saccharomyces* yeast strains into useful model systems for research in ecology and evolution. Yet, the variation between species and strains in important population parameters remains largely undescribed. Here, we investigated the relationship between two commonly used measures in microbiology to estimate growth rate – cell density and cell count – in 23 strains across all eight *Saccharomyces* species *.* We found that the slope of this relationship significantly differs among species and a given optical density (OD) does not translate into the same number of cells across species. We provide a cell number calculator based on our OD measurements for each strain used in this study. Surprisingly, we found a slightly positive relationship between cell size and the slope of the cell density-cell count relationship. Our results show that the strain- and species-specificity of the cell density and cell count relationship should be taken into account, for instance when running competition experiments requiring equal starting population sizes or when estimating the fitness of strains with different genetic backgrounds in experimental evolution studies.

**Figure 1. Relationship between OD (optical density), cell count, and cell size:**
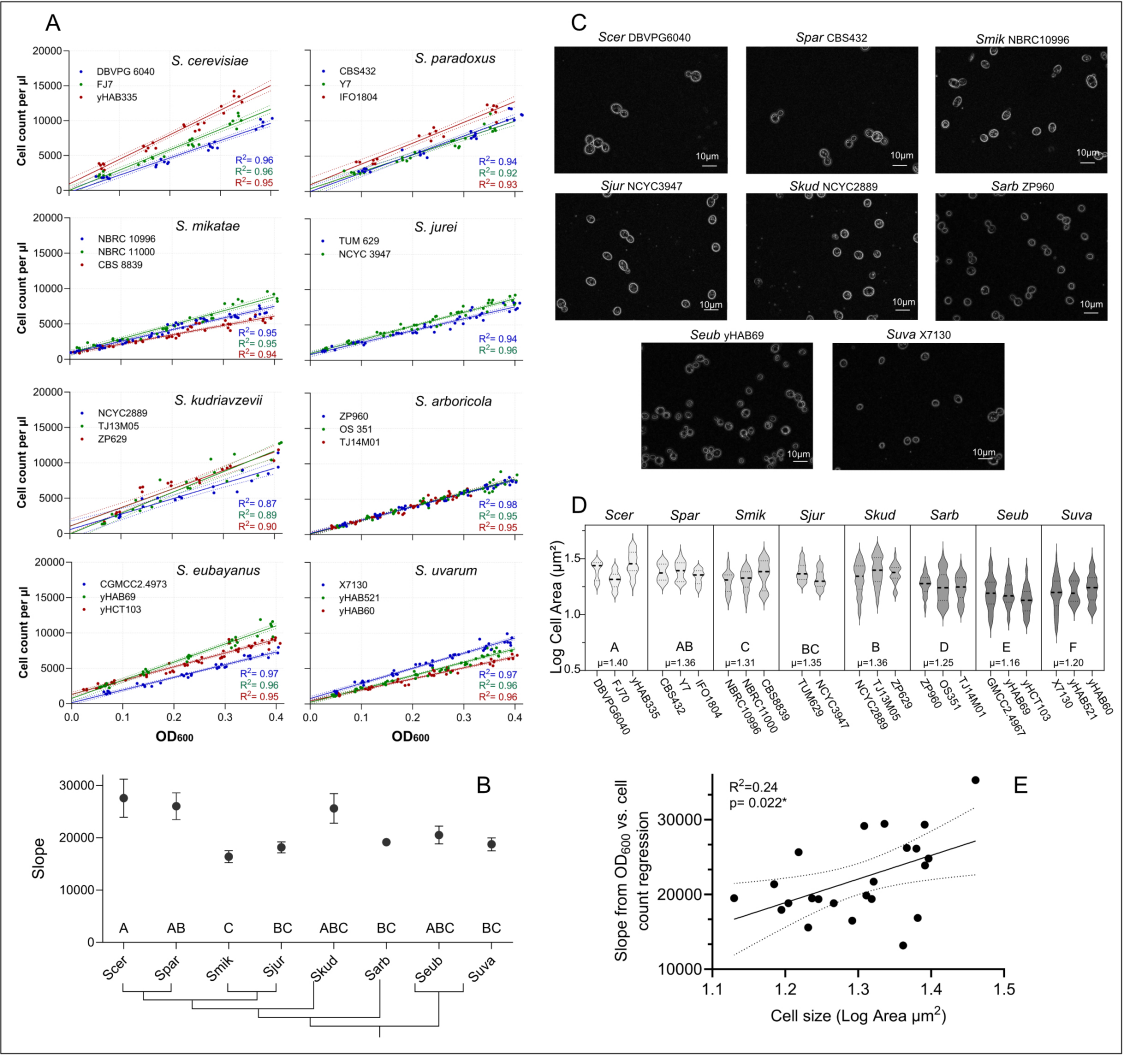
**A)** Linear regression of cell count (obtained from flow cytometry as number of cells/μL) on optical density (obtained from spectrophotometry as OD _600_ ) across eight *Saccharomyces* species. Coloured lines represent three genetically different strains per species (except for *S. jurei* , where only 2 strains were available). Strain names are indicated on each species’ plot. Dotted lines are 95% confidence intervals. **B)** Slopes of eight *Saccharomyces* species extracted from regressions of cell counts (obtained from flow cytometry) on optical density (OD _600_ ). Slopes are averaged across strains within species and expressed as the cell count increasing per OD _600_ unit. Error bars represent 95% confidence intervals from regression of all data per species. Levels not connected by same letter are significantly different. The species’ phylogenetic relationship is shown at the bottom. **C** ) Images of yeast cells at 100x magnification. **D)** Violin plot showing the distribution of cell size (log cell area in µm ^2^ ). Average cell size per species is shown at the bottom. The darker the violin plots, the smaller cells are on average. Levels not connected by same letter are significantly different in pairwise Tukey HSD tests after ANOVA of ‘species’ on ‘log cell area’ (F _7,1710_ = 96.12, p < 0.001). **E)** Scatter plot of slopes from linear regressions of cell count (obtained from flow cytometry as number of cells per μL) on optical density (obtained from spectrophotometry as OD _600_ ) against average cell size (log cell area in µm ^2^ ) per strain.

## Description

The genus *Saccharomyces* (Saccharomycetales, Ascomycota) originated ~100 million years ago facilitated by whole genome duplication (Wolfe & Shields, 1997) . It currently comprises eight genetically different species that range in divergence times from 4 to 20M years (Borneman & Pretorius, 2014; Kellis et al., 2003; Shen et al., 2018) *.* All species are monophyletic with high levels of sequence collinearity (Bendixsen et al., 2021; Liti et al., 2006) but show vast genetic and ecological diversity (Boynton & Greig, 2014) . Recently, the genomic and ecological data available for all eight species (and their hybrids) have considerably grown (Bendixsen et al., 2022) , promoting yeast from a traditional laboratory model system that only included a few clonal strains to one in which we can answer questions relevant for ecology and evolution (Stelkens & Bendixsen, 2022) . Our growing knowledge of the phenotypic and genetic diversity of wild yeasts also allows us to study important ecological traits comparatively, across species backgrounds, alleviating limitations resulting from only including a handful of well-characterized laboratory strains in our analyses. For instance, one ecological trait that stands out as particularly diverse between species are their temperature preference profiles (Abrams et al., 2021; Baker et al., 2019; Gonçalves et al., 2011; Salvadó et al., 2011; Weiss et al., 2018). Of the eight species included here, five are considered cold-tolerant ( *S. kudriavzevii, S. arboricola, S. uvarum* , *S. eubayanus* , and the recently discovered *S. jurei)* , one species is thermo-tolerant ( *S. cerevisiae* ), and two species are considered a thermo-generalist ( *S. paradoxus* and *S. mikatae* ) growing well in a broad range of temperatures (Libkind et al., 2011; Naseeb et al., 2017; Robinson et al., 2016; Salvadó et al., 2011). Since resistance and adaptation to high or fluctuating temperatures are an increasing research focus for climate change biology, we consider it important to better characterise the biological features of a larger range of wild yeast strains and species before we use them as model systems.

Developing wild strains into effective systems for research and industry requires the systematic testing and measuring of fundamental population phenotypes including their growth rates, kinetics (e.g. the length of the lag phase), and yield. Microbial research often applies high-throughput methods to estimate population growth and fitness in environments of interest, e.g. media containing different nutrients and stress conditions. A common technique is to measure the optical density (OD) of microbial cultures using a spectrophotometer (‘plate reader’). Optical density measures the turbidity of liquid cultures, which is assumed to be proportional to the cell number, i.e. the concentration of cells in the sample (Stevenson et al., 2016) . Specifically, OD is the negative log of transmittance, i.e. the fraction of light detected when passed through a cuvette or micro-titer plate containing the microbial culture. It is typically measured at a wavelength of 600nm as this electromagnetic radiation is thought to not cause cell damage. Calculations follow the Beer-Lambert law (Mayerhöfer et al., 2020; Swinehart, 1962), which states that OD is proportional to the concentration of a solution. However, this law only applies to cultures with low cell densities (typically OD _600_ up to 0.1). At higher densities, the light gets increasingly scattered between cells, and OD does not increase as fast as the cell titer. Using spectrophotometry to infer population fitness has additional limitations affecting the translation of OD into cell counts. Importantly, the method does not differentiate between dead and alive cells and the absorption coefficient (ε) can be affected by cell size (Fukuda 2023) . Different methods exist for the calibration of OD measurements (Stevenson et al., 2016) , including the use of silica microspheres, direct cell counting with microscopy, and colony counting in serial dilutions on agar plates (Beal et al., 2020) . But the most efficient, high-throughput method is flow cytometry, which uses laser-based detection of individual cells to allow for accurate cell count estimates (Boyd et al., 2000, 2006; Jahan-Tigh et al., 2012) .

Here, we explore the variation in the relationship between OD measures from spectrophotometry and cell counts from flow cytometry, across all eight *Saccharomyces* yeast species. To also test for variation within species, we used three strains per species (except for *S. jurei* , where only two strains were available) from different geographic and ecological origins, including isolates from fruit, soil, rotten wood, and tree bark from Europe, Asia, North and South America, and Australia. Our aim is to expand the knowledge base of important growth parameters of non- *cerevisiae* strains and to improve the biological interpretation of population fitness data from wild, non-domesticated yeasts.

***The relationship between optical density and cell count differs between species***

To investigate the relationship between cell count (obtained from flow cytometry) and OD _600_ -value (from spectrophotometry) across all 23 strains, we used linear regression ( Figure 1A, all R ^2^ > 0.87, *p* < 0.01). We found significant differences between species in the slope of this relationship (ANOVA: F _7,22_ = 5.63, p = 0.0025; Figure 1B ). The average slope extracted from *S. cerevisiae* regressions was significantly steeper than the slopes found in *S. mikatae,* *S. jurei, S. arboricola* , and *S. uvarum* (Tukey HSD, all *p* < 0.05), i.e. *S. cerevisiae* produces higher cell counts at lower OD values. The random effect ‘strain’ was non-significant and only explained 4% of the variation in the data overall (Wald *p* = 0.81).

The steepness of the cell density – cell count relationship is informative for researchers conducting studies across species and strain backgrounds. We provide a simple open-access tool to convert OD measurements into cell numbers for each of the strains used in this study here: https://tinyurl.com/8sn2y2b4 . Interestingly, the patterns in slope variation that we observed across the genus, closely follow the phylogenetic relationship of the species, with more closely related pairs ( *S. cerevisiae* / *S paradoxus* , *S. mikatae* / *S. jurei* , and *S. eubayanus* / *S. uvarum* ) having more similar slopes ( Figure 1B ). This suggests that the species- and strain-specificity of this relationship should be taken into account when setting up and interpreting results of competitive fitness assays, or when fine-tuning cell titers and inocula of non-commercial strains for industrial applications. It also shows the limitations of using only a single laboratory *S. cerevisiae* strain as a reference point when exploring the population biology of wild strains.

***Yeast species significantly differ in cell size***

Variation in cell size between yeast species is expected, given the large diversity of ecological niches these species inhabit, and has been described in *S. cerevisiae* as a result of temperature (Zakhartsev & Reuss, 2018) and nutrient availability (Kellogg & Levin, 2022) , affecting basic physiological functions such as protein synthesis and cell division rate (Schmoller & Skotheim, 2015; Turner et al., 2012) . Indeed, we found that species significantly differed in average cell size (log cell area in µm ^2^ ) (mixed model output for fixed effect ‘species’: F _7_ = 14.17, p < 0.0001; Figure 1C, 1D). Overall, *S. eubayanus* have the smallest (1.16 ± 0.12 log µm ^2^ ) and *S. cerevisiae* have the largest cells (1.4 ± 0.11 log µm ^2^ , Figure 1D . *S. arboricola* , *S. eubayanus,* and *S. uvarum* cells are on average significantly smaller than the cells of all other species, while *S. cerevisiae* and *S. paradoxus* have significantly larger cells than most other species ( Figure 1C ). The random effect ‘strain’ explained a small (7.1%) but significant proportion of the variance in cell size (Wald *p* = 0.041). Indeed, most species showed some significant differences in cell size between strains (ANOVA for all species/t-test for *S. jurei* : all F/t > 3.40, all *p* < 0.04), except for the three *S. arboricola* strains, which were more uniform in size.

***Cell size explains variation in the cell density - cell count relationship***

We speculated that species differences in cell size may explain variation in the steepness of the slopes extracted from cell density - cell count regressions, because the intensity and radius of light scattering in the spectrophotometer are known to depend on cell size, which affects the absorbance of the microbial culture (Fukuda, 2023; Stevenson et al., 2016) . We expected cell size to inversely predict the steepness of slopes, because a given optical density may result in fewer cell counts if cells are on average larger. However, we found the opposite in our data: the steepness of strain-specific slopes extracted from cell density - cell count regressions increased slightly with increasing strain cell size (R ^2^ = 0.24; *p* = 0.0223; Figure 1E ), suggesting that at a given OD-value, strains with larger cells also produce higher cell counts.

Besides cell size, other species-specific cellular features can affect the cell count – cell density relationship. The species investigated here are ecologically and genetically vastly divergent and likely differ in the composition of their cell wall, determining their strength and rigidity (Brown & Esher, 2020; Nguyen et al., 1998) . Wall-resident proteins have diversified rapidly over evolutionary time in *Saccharomyces* as a result of gene silencing through epigenetic mechanisms and environmentally induced expression regulation, providing adaptability to different habitats and lifestyles (Lozančić et al., 2021; Xie & Lipke, 2010). Besides cell wall composition, the number and structure of bud scars may also affect the cell’s refractive index (Chaudhari et al., 2012) . Mother cells accumulate chitinous scar tissue from cytokinesis over their lifetime (Powell et al., 2003) and different growth conditions (poor *vs* . rich media) can lead to variation in bud scar number (Lorincz & Carter, 1979) . If species vary in the average number or structure of bud scars a mother cell carries, e.g. due to heritable differences in cell longevity or species-specific responses to nutrient availability. High flocculation results in a decrease in the optical density (Smit et al., 1992) . In this study, we used non-domesticated strains that did not show evident flocculation; nevertheless, microscopy revealed a certain level of clumping, being more evident in *S. eubayanus* yHAB69 and *S. paradoxus* CBS432. Future studies may investigate the impact of cell composition and bud scars on the refractive indices of cells and how these factors may affect the translation of optical density readings into cell counts.

## Methods

***Selection of strains***

We selected a total of 23 strains. Each of the eight *Saccharomyces* species is represented by three strains isolated from different geographic locations and habitats, with the exception of the newly discovered *S. jurei* , for which only two strains were available to us. We only used non-domesticated, wild-type strains that do not show flocculation or cell clumping.

***Cell density and cell count measurements***

We grew yeast strains from frozen glycerol stocks overnight in 5 mL of YPD liquid (Yeast Peptone Dextrose) and then transferred 100 ul to YNB complete medium (0.67% Yeast Nitrogen Base; 2% glucose). The culture was incubated at 25°C during 12 hours. OD _600_ was measured with a spectrophotometer (BioTek Epoch 2) in 96-well plates with 200 µL yeast culture per well, in four dilutions with miliQ water (2, 2.5, 3.33, 5, 10 and 20-fold dilution) to provide a range of OD readings. Raw OD measurements were blank-corrected. The average OD of each strain was calculated from six technical replicates per dilution, with three independent read-outs obtained from the plate reader. To maintain linearity between cell number and OD _600_ values, OD must be within the dynamic range of equipment. We therefore used an OD _600_ of 0.4 as the upper limit for cell counting.

For flow cytometry, we used the same cultures that we had prepared for the OD _600_ measurements, and diluted them further in phosphate-buffered saline (PBS) with a 50X dilution factor in 96 well plates. The plate layout, i.e. the position of strains and replicates on the 96-well plates, were identical in both the spectrophotometer and flow cytometry runs.

Linear regressions between cell density (OD from spectrophotometry) and cell count (from flow cytometry) were performed. Goodness of fit (R ^2^ ) and 95% confidence intervals were calculated and plotted along the slope in GraphPad Prism 10. Species and strain-specific slopes were extracted as the change of Y per one unit increase in X (i.e. the cell count increasing per OD _600_ unit). We tested for differences between species in slopes, using ANOVA with Tukey HSD tests for pairwise comparisons in JMP (v17.2.0). To assess how much of the variance in slope is explained by differences between strains, we applied a mixed model using ‘species’ as fixed and ‘strain’ as random effect.

***Cell size measurements***

Strains were grown from frozen glycerol stocks overnight in 5 mL liquid YNB complete medium, and diluted using a 50X dilution factor. Cell photos were taken with a compound microscope (Leica ^TM^ ) at 100X magnification connected to a DSRL camera. Between 69 and 364 cells were measured per species (including all strains per species). Photos were processed using the StarDist plugin for ImageJ/Fiji software, a cell detection method for microscopy images, and processed using ImageJ/Fiji’s ‘find edges’ option. We used the detection model DSB 2018 (Schmidt et al., 2018) , setting the probability/score threshold to 80 and the overlap threshold to 70. Cell area was measured and log-transformed and a mixed model using ‘species’ as fixed effect and ‘strain’ as random effect was used on cell size as a response variable in JMP (v17.2.0).

***Cell count calculator***

We obtained the equations from linear regression of OD and cell number of each of the 23 strains used in this study (cell count=m*OD+b) to provide a simple, open-access conversion tool that uses OD values (x) as a predictor of cell count (y): https://tinyurl.com/8sn2y2b4 .

## Reagents

**Table:** 

**Species**	**Strain**	**Location**	**Reference**
*S. cerevisiae*	NCYC 3557	Netherlands	Camarasa et al., 2011
	FJ7	Fujian, China	Wang et al., 2012
	yHAB335	Shannxi, China	unknown
*S. paradoxus*	CBS432	Russia	Bachinskaya, 2014
	Y7	London, UK	Cubillos et al., 2009
	IFO1804	Japan	Naumov et al., 1996
*S. mikatae*	NBRC 10996	Nagano, Japan	NITE Biological Resource Center, Chiba, Japan
	NBRC 11000	Iwate, Japan	NITE Biological Resource Center, Chiba, Japan
	CBS 8839	Japan	Naumov et al., 2000
*S. jurei*	TUM 629	Munich, Germany	Hutzler et al., 2021
	NCYC 3947	Saint Auban, France	Naseeb et al., 2017
*S. kudriavzevii*	NCYC2889	Japan	Naumov et al., 2000
	TJ13M05	Taiwan	Naumov et al., 2013
	ZP629	Portugal	Sampaio & Gonçalves, 2008
*S. arboricola*	ZP960	New Zealand	unknown
	OS 351	Yunnan, China	Wang & Bai, 2008
	TJ14M01	Shaanxi, China	Wang & Bai, 2008
*S. eubayanus*	CGMCC2.4973	Sichuan, China	Bing et al., 2014
	yHAB69	Puyehue, Argentina	Eizaguirre et al., 2018
	yHCT103	Ñirihuau, Argentina	Eizaguirre et al., 2018
*S. uvarum*	X7130	Germany	unknown
	yHAB521	South America	Sylvester et al., 2015
	yHAB60	South America	Eizaguirre et al., 2018
